# Editorial: Exercise intervention for prevention and management of hypertension

**DOI:** 10.3389/fphys.2023.1244715

**Published:** 2023-07-04

**Authors:** Aline Mendes Gerage, Cristine Lima Alberton, Gabriel Grizzo Cucato, Rodrigo Sudatti Delevatti, Raphael Mendes Ritti-Dias

**Affiliations:** ^1^ Department of Physical Education, Federal University of Santa Catarina, Florianopolis, Brazil; ^2^ Department of Sports, Federal University of Pelotas, Pelotas, Brazil; ^3^ Department of Sport, Exercise and Rehabilitation, Northumbria University, Newcastle upon Tyne, United Kingdom; ^4^ Post-Graduate Program in Rehabilitation Sciences, Nove de Julho University, São Paulo, Brazil

**Keywords:** hypertension, exercise, physical activity, prevention, intervention-behavioral

Hypertension is a significant global public health issue. Exercise has long been regarded as a fundamental component in the prevention and treatment of hypertension (Pescatello et al., 2015). Daily physical activities, aerobic and resistance training programs have consistently been studied in the hypertensive population, and their effectiveness in enhancing cardiovascular and physical function has been well-described (Gerage et al., 2015; Pescatello et al., 2015; MacDonald et al., 2016).

The Research Topic “*Exercise intervention for the prevention and management of hypertension*” presents information on the cardiovascular effects of various forms of exercise through original and review articles. The exercises analyzed include deep breathing exercises, Liuzijue training, and inspiratory muscle training. Additionally, the influence of leisure time resistance activities and the variability of 24-h acute blood pressure responses to exercise are examined. All these studies provide different insights into the therapeutic effects of exercise in individuals with hypertension.

The therapeutic role of deep breathing exercises, which is a broad term encompassing several types of non-resisted, paced breathing strategies such as yogic breathing or Pranayama, diaphragmatic breathing, and abdominal breathing, in reducing blood pressure was discussed in two literature reviews. Tavoian and Craighead explored the use of deep breathing exercises in work settings as a strategy to reduce blood pressure levels and manage work-related stress. They summarized the physiological and psychological effects of deep breathing to support its implementation in the workplace, which is often characterized by frequent episodes of mental health conditions. The authors presented promising real-world evidence that supports the plausibility of deep breathing interventions in this context.

The scoping review conducted by Herawati et al. summarized the currently available information on the advantages of breathing exercises in decreasing blood pressure in patients with hypertension. The review included 20 studies with a mean PEDro score of 7.4, in which resting blood pressure and heart rate were the most frequently assessed outcomes. The results of the studies exhibited wide variability, with systolic blood pressure declining by 4–54.22 mmHg, and diastolic blood pressure dropping by 3–17 mmHg with deep breathing exercises. Based on the available evidence, it is suggested that this exercise strategy holds merit in improving cardiovascular parameters in hypertensive patients. However, further studies are warranted to understand the optimal exercise protocols and the underlying mechanisms behind these effects.

The original article developed by Carpes et al. aimed to measure inter-individual variations in 24-h blood pressure after a single bout of various exercise modalities in older adults with hypertension. They investigated the mean changes in 24-h blood pressure in participants with hypertension after aerobic, combined, resistance, and isometric handgrip exercise sessions related to a non-exercising control session. The prevalence of responders in the total 24-h ranged from 22% to 37% for systolic and from 26% to 53% for diastolic blood pressure after different exercise modalities. Authors concluded that various exercise modalities might acutely reduce 24-h blood pressure in older adults with hypertension, although a high inter-individual variability has been observed. These responses are important because the chronic hypotensive effect of physical training appears to be related to the sum of the acute blood pressure reduction that occurs after an exercise session.

Evaluating the effects of inspiratory muscle training on the blood pressure of patients with hypertension, Zheng et al. conducted a systematic review with meta-analysis, founding important results. This review summarized findings from eight randomized controlled trials totaling 215 patients. Expressive alterations were found in some cardiovascular outcomes, highlighting a reduction of 12.55 mmHg (95% CI: −15.78, −9.33) and 4.77 mmHg (95% CI: −6.00, −3.54) in systolic and diastolic blood pressure, respectively. In addition, a subgroup analysis showed superior blood pressure reductions in low intensity IMT compared to medium-high intensity inspiratory muscle training. Even with positive cardiovascular findings, the authors presenting inspiratory muscle training as an auxiliary approach in the clinical exercise context for hypertension management.

A study conducted by Wu et al. aimed to compare the effectiveness of Liuzijue training (a Chinese traditional exercise that combines breathing meditation and aerobic exercise) with traditional aerobic training on blood pressure, endothelial function, and gut microbiota composition in hypertensive patients. Results showed that Liuzijue training was more effective in lowering blood pressure levels in 57% of participants, compared to 25% in the traditional aerobic training group. Liuzijue training also appeared to improve immune homeostasis by reducing pro-inflammatory markers and promoting a healthier gut microbiota balance. The study suggests a potential link between immune factors and gut microbiota, which could be the mechanism behind Liuzijue training’s ability to lower blood pressure levels. However, further research is necessary due to the limited patient sample size.

Finally, Park et al. analyzed the role of physical activity and the regular practice of resistance exercise in the prevention of hypertension in a community-based cohort of 5,075 adults. After a follow-up of almost 8 years on average, it was found that those who had high levels of physical activity decreased their risk of developing hypertension in 30% compared to those with low levels of physical activity. The addition of the regular practice of resistance exercise to high levels of physical activity further prevented the development of hypertension only in women, while the practice of resistance training alone, without satisfactory leisure-time physical activity did not affect the incidence of hypertension. This study emphasizes the importance of meeting the global recommendations of physical activity and the relevant potential of resistance training in cardiovascular prevention. Future studies are required to investigate the sex differences and training intensity in resistance training related antihypertensive effects.

Overall, the research discussed in this Research Topic underscores the importance of exercise as a major component in preventing and managing hypertension. It highlights the effectiveness of various exercise strategies in improving cardiovascular parameters, reducing blood pressure, and potentially preventing the development of hypertension.
